# A novel swine model of the acute respiratory distress syndrome using clinically relevant injury exposures

**DOI:** 10.14814/phy2.14871

**Published:** 2021-05-15

**Authors:** Mohamad H. Tiba, Brendan M. McCracken, Danielle C. Leander, Carmen I. Colmenero, Jean A. Nemzek, Michael W. Sjoding, Kristine E. Konopka, Thomas L. Flott, J. Scott VanEpps, Rodney C. Daniels, Kevin R. Ward, Kathleen A. Stringer, Robert P. Dickson

**Affiliations:** ^1^ Department of Emergency Medicine University of Michigan Ann Arbor MI USA; ^2^ Michigan Center for Integrative Research in Critical Care University of Michigan Ann Arbor MI USA; ^3^ Unit of Laboratory Animal Medicine University of Michigan Ann Arbor MI USA; ^4^ Division of Pulmonary and Critical Care Medicine Department of Internal Medicine University of Michigan Ann Arbor MI USA; ^5^ Institute for Healthcare Policy and Innovation University of Michigan Ann Arbor MI USA; ^6^ Department of Computational Medicine and Bioinformatics University of Michigan Ann Arbor MI USA; ^7^ Department of Pathology University of Michigan Ann Arbor MI USA; ^8^ Department of Clinical Pharmacy College of Pharmacy University of Michigan Ann Arbor MI USA; ^9^ Department of Biomedical Engineering University of Michigan Ann Arbor MI USA; ^10^ Biointerfaces Institute University of Michigan Ann Arbor MI USA; ^11^ Department of Pediatrics Pediatric Critical Care Medicine University of Michigan Ann Arbor MI USA; ^12^ Department of Microbiology & Immunology University of Michigan Ann Arbor MI USA

**Keywords:** acute lung injury, acute respiratory distress syndrome, critical care, diffuse alveolar damage, direct lung injury, indirect lung injury, porcine models, sepsis, septic shock

## Abstract

To date, existing animal models of the acute respiratory distress syndrome (ARDS) have failed to translate preclinical discoveries into effective pharmacotherapy or diagnostic biomarkers. To address this translational gap, we developed a high‐fidelity swine model of ARDS utilizing clinically relevant lung injury exposures. Fourteen male swine were anesthetized, mechanically ventilated, and surgically instrumented for hemodynamic monitoring, blood, and tissue sampling. Animals were allocated to one of three groups: (1) *Indirect lung injury only*: animals were inoculated by direct injection of *Escherichia coli* into the kidney parenchyma, provoking systemic inflammation and distributive shock physiology; (2) *Direct lung injury only*: animals received volutrauma, hyperoxia, and bronchoscope‐delivered gastric particles; (3) *Combined indirect and direct lung injury*: animals were administered both above‐described indirect and direct lung injury exposures. Animals were monitored for up to 12 h, with serial collection of physiologic data, blood samples, and radiographic imaging. Lung tissue was acquired postmortem for pathological examination. In contrast to *indirect lung injury only* and *direct lung injury only* groups, animals in the *combined indirect and direct lung injury* group exhibited all of the physiological, radiographic, and histopathologic hallmarks of human ARDS: impaired gas exchange (mean PaO_2_/FiO_2_ ratio 124.8 ± 63.8), diffuse bilateral opacities on chest radiographs, and extensive pathologic evidence of diffuse alveolar damage. Our novel porcine model of ARDS, built on clinically relevant lung injury exposures, faithfully recapitulates the physiologic, radiographic, and histopathologic features of human ARDS and fills a crucial gap in the translational study of human lung injury.

## Graphical abstract

Our novel porcine model of the acute respiratory distress syndrome, built on clinically‐relevant lung injury exposures, faithfully recapitulates the physiologic, radiographic, and histopathologic features of human ARDS, and fills a crucial gap in the translational study of human lung injury.

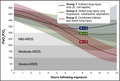

## 1 INTRODUCTION

The acute respiratory distress syndrome (ARDS) is a life‐threatening lung condition that affects more than 200,000 people in the United States each year with a mortality rate of approximately 40% (Erickson et al., (2009); Robles et al., 2018). As a clinically defined syndrome, ARDS has undergone only modest refinement in its definition since its first report in 1967 (Ashbaugh et al., 1967; Bernard et al., 1994; Ranieri et al., 2012). Despite a half century of experimental and clinical study, the diagnosis of ARDS remains entirely clinical (with no molecular biomarkers), and its management remains entirely supportive (with no targeted therapies).

A major barrier to advances in our diagnosis and management of ARDS has been reliance on inadequate preclinical animal models to study the syndrome (Semler et al., 2020). The vast majority of experimental research on ARDS has been performed using small animal (i.e., rodent) models (Matute‐Bello et al., 2011). This reliance on rodent modeling of ARDS has not been due to their fidelity to human disease but rather due to ease of handling, cost, accessible reagents, and availability of purebred and genetically engineered strains. Anatomically, murine lungs have a distinct lobar structure with far fewer branching airways than humans, extensive bronchial‐associated lymphoid tissue, and a near‐absence of submucosal glands (Matute‐Bello et al., 2011). Mice also profoundly differ from humans in their innate and adaptive immune response to injury, including fewer circulating neutrophils, absence of defensins, and a distinct chemokine repertoire (Mestas & Hughes, 2004). Notably, murine lungs almost never form hyaline membranes, a histopathological hallmark of diffuse alveolar damage (the histopathological hallmark of human ARDS) (Matute‐Bello et al., 2011). For these reasons, the 2011 American Thoracic Society workshop report on experimental acute lung injury conceded “the responses of animal [murine] and human lungs to an injurious stimulus cannot be expected to be identical or perhaps even similar.”(Matute‐Bello et al., 2011). Additionally, rodent models almost all preclude the study of co‐interventions and organ support (e.g., vasopressors or mechanical ventilation), serial sampling across anatomic compartments, or radiographic study. For these reasons, the NHLBI has identified the need for large‐animal models of ARDS as a research priority (Semler et al., 2020).

In contrast to rodent models, swine models of ARDS represent a promising alternative. The swine lung exhibits lobar, interlobular, and airway anatomy similar to that of humans (Judge et al., 2014), and immune gene expression of swine is far more similar to that of humans (Bailey et al., 2013; Dawson et al., 2013, 2017; Merrifield et al., 2011; Meurens et al., 2012; Meyerholz et al., 2016; Wernersson et al., 2005). The metabolite composition of swine lung tissue is far more representative of human lungs than is that of rodent species (Merrifield et al., 2011). Several swine models of ARDS exist, yet these rely on clinically unrepresentative single exposures (e.g., oleic acid infusion [Dickson et al., 2011; Schuster, 1994] and surfactant washout [Russ et al., 2016]). To our knowledge, no existing swine model recapitulates the core features of human ARDS using clinically relevant exposures.

To address these gaps, we sought to establish a preclinical model of ARDS using clinically relevant exposures that (1) faithfully recapitulates the physiologic, radiographic, and histopathologic features of human ARDS, (2) allows for longitudinal study of pathogenesis, underlying mechanisms, and treatment strategies, and (3) permits study of co‐interventions and organ support (e.g., vasopressors and mechanical ventilation). Motivated by clinical (Sjoding et al., 2019) and experimental (Wiener‐Kronish et al., 1991) observations that both epithelial and endothelial injury are necessary to provoke the full pathophysiologic and clinical manifestations of ARDS, we hypothesized that a combination of indirect lung injury (sepsis [Tiba et al., 2020]) and direct lung injury (concurrent administration of volutrauma, hyperoxia, and instillation of gastric particles into the airways) would be required to induce all of the clinical and biological hallmarks of human ARDS. Selective data from the *indirect lung injury only* (sepsis) group have previously been published (Tiba et al., 2020).

## 2 METHODS

This study adhered to the principles stated in the *Guide for the Care and Use of Laboratory Animals* (National Research Council (U.S.) Committee for the Update of the Guide for the Care and Use of Laboratory Animals, Institute for Laboratory Animal Research (U.S.), and National Academies Press (U.S.), 2011) and was approved by the Institutional Animal Care and Use Committee (IACUC). Animals were acquired through an IACUC‐approved vendor and acclimated for 5–10 days before experimentation.

### 2.1 Anesthesia and surgical instrumentation

Fourteen male Yorkshire‐mix swine, approximately 14–16 weeks of age, were fasted overnight with ad libitum access to water. Anesthesia was induced using an intramuscular injection of ketamine/xylazine combination (22 and 2 mg/kg) followed by 1.5%–2.5% isoflurane administered through a facemask. Animals were intubated using a 7.5‐mm cuffed endotracheal tube and mechanically ventilated to maintain end‐tidal CO_2_ (PetCO_2_) at 35–45 mmHg. Heart rate (HR), electrocardiograph (ECG), PetCO_2_, and pulse oximeter oxygen saturation (SpO_2_) were monitored using a veterinary monitor (Surgivet advisor, Smiths Medical). Body temperature was maintained between 37°C and 38.5°C using a feedback‐controlled warming blanket (Cincinnati SubZero, Blanketrol II).

Under aseptic conditions, the right carotid artery and the right external and internal jugular veins were cannulated to provide continuous monitoring of mean arterial blood pressure (MAP), pulmonary artery pressure (PAP), heart rate, core temperature, and arterial and mixed venous blood sampling as well as for intravenous anesthetic administration. A midline laparotomy was performed to access the bladder, the left kidney, isolate the ureter, and for placement of an indwelling Foley catheter for urine draining. At the end of surgical instrumentation, inhalant anesthesia was transitioned to total intravenous anesthesia by continuous infusions of midazolam (5–20 mcg/kg/min), fentanyl (0.03–4.0 mcg/kg/min), and propofol (10–100 mcg/kg/min) for the remainder of the experiment. Baseline hemodynamic metrics and blood samples for hematology, serum chemistry, and arterial and venous lactate, glucose, electrolytes, oximetry, and blood gasses were obtained (Table 1). Ventral‐dorsal thoracic radiographic images were obtained using a veterinary portable X‐ray (MinXray hf100+, MinXray). A 5‐ml inoculum (*Escherichia* *coli* culture or saline sham) was administered into the left kidney parenchyma over 15 min (0.33 ml/min), and postinjection procedures were done as previously described (Tiba et al., 2020). Completion of the renal inoculation was considered Time 0 (*T*_0_). The abdominal wall and skin were closed in layers. The ureter was occluded for a duration of 1 h and then unoccluded.

**TABLE 1:** Baseline characteristics by group

Characteristic	Experimental group		
	Group 1: *Indirect lung injury only*	Group 2: *Direct lung injury only*	Group 3: *Combined indirect and direct lung injury*
*n*	5	4	5
Weight (kg)	43 (3.81)	44 (2.06)	43 (0.71)
Mean arterial pressure (mmHg)	85.5 (9.31)	90.1 (15.13)	93.0 (7.19)
Pulmonary artery pressure (mmHg)	21.3 (7.38)	13.5 (6.23)	18.6 (2.90)
Heart rate (BPM)	74 (5.8)	78 (6.5)	70 (9.3)
Temperature (°C)	37.8 (0.80)	37.2 (0.84)	37.4 (0.61)
pH	7.474 (0.022)	7.401 (0.081)	7.442 (0.093)
Lactate (mEq/L)	1.4 (0.95)	0.5 (0.05)	0.7 (0.36)
SaO_2_ (%)	98.2 (1.28)	100 (0.00)	100 (0.00)
SmvO_2_ (%)	58.8 (1.92)^a^, ^b^	75.2 (6.26)	78.8 (4.62)
PaO_2_/FiO_2_ ratio	470 (47.9)	454 (28.8)	494 (67.4)
PetCO_2_ (mmHg)	43.9 (3.92)	39.9 (2.15)	39.0 (3.69)
PaCO_2_ (mmHg)	40.4 (5.01)	46.3 (6.76)	43.7 (6.68)
White blood count (10^9^/L)	17.06 (3.62)	17.65 (5.41)	14.67 (2.16)
Monocytes (10^9^/L)	0.16 (0.104)	0.11 (0.063)	0.09 (0.039)
Lymphocytes (10^9^/L)	10.41 (2.420)	10.98 (2.354)	8.57 (1.210)
Neutrophils (10^9^/L)	6.48 (2.270)	6.56 (3.363)	6.00 (1.276)
Blood urea nitrogen (mg/dl)	7.4 (3.43)	5.5 (1.29)	7.6 (2.40)
Creatinine (mg/dl)	1.3 (0.11)^a^	1.0 (0.14)	1.2 (0.19)
Hematocrit (%)	40.6 (6.55)	38.1 (2.83)	35.6 (3.07)
Platelet (10^9^/L)	279 (102.3)	294 (44.0)	283 (97.6)

Note

Data are presented as mean (standard deviation). Statistical significance was set at *α* < 0.05. PaO_2_, Partial pressure of oxygen. FiO_2_, fraction of inspired oxygen. SmvO_2_, mixed venous oxygen saturation (%); PetCO_2_, end‐tidal CO_2_ (mmHg). SaO2, arterial oxygen saturation. PaCO_2_, partial pressure of arterial CO_2_.

a Statistically significant difference between Group 1 and Group 2.

b Statistically significant difference between Group 1 and Group 3.

To ensure humane experimentation, our protocol included prespecified criteria for experiment termination: (1) persistent low mean arterial pressure (<40 mmHg) for more than 2 h, (2) persistent low PetCO_2_ (<25 mmHg) for more than 2 h, (3) critical low mean arterial pressure (<25 mmHg) combined with critical low PetCO_2_ (<20 mmHg), (4) critical low PaO_2_ (<55 mmHg) for more than 1 h, (5) ventricular fibrillation/tachycardia, and (6) malignant hyperthermia due to inhalant anesthetics.

To standardize temporal comparisons, primary physiologic, radiographic, and histopathologic comparisons were made at hour 12 following exposure. Lung tissue was collected at the time of experiment termination, which was 12 h following exposure for most animals. Three animals met termination criteria prior to hour 12 (detailed in Results). One animal each in Group 1 (*Indirect lung injury only*) and Group 2 (*Direct lung injury only*) was observed for 19 h to establish trajectory. For these animals, lung tissue was collected at the time of experiment termination. The PaO_2_/FiO_2_ ratio for these animals at hour 19 was 334 and 262, respectively.

### 2.2 Experimental groups and exposures

Animals were designated into one of three experimental groups as follows. The assignment of animal to experimental group was not randomized.

#### 2.2.1 Group 1—*indirect lung injury only*

As recently described (Tiba et al., 2020), a total volume of 5 ml containing an average culture count of 2.5 × 10^11^ CFUs of live *E*. *coli* was administered into the kidney's parenchyma. No antibiotics were administered. Tidal volume (Vt) was set between 7 and 8 ml/kg using 21% fractional inspired O_2_ (FiO_2_) and 5 cmH_2_O of positive end‐expiratory pressure (PEEP). A restrictive (minimal) fluid resuscitation strategy was used (30 ml/h normal saline). As previously reported (Tiba et al., 2020), this exposure provokes systemic inflammation and distributive shock physiology characteristic of sepsis.

#### 2.2.2 Group 2—*direct lung injury only*

(1) Volutrauma: Tidal volume was set between 12 and 15 ml/kg during instrumentation and continued for the duration of the experiment. PEEP was set at 0 cmH_2_O. (2) Hyperoxia: FiO_2_ was set to 100% during instrumentation and continued for the duration of the experiment. (3) Instillation of gastric particles into the lungs: Prior to experiments, a uniform stock of gastric contents from donor pigs was homogenized in sterile saline, strained, filtered, and autoclaved. Briefly, a uniform stock of gastric contents was prepared from donor pigs euthanized at the conclusion of other studies. The intact stomachs of pigs fed standard laboratory chow were removed immediately postmortem. Stomach contents were collected and homogenized in sterile saline and strained using sterile cotton gauze. The filtered contents were passed through a 200‐µm nylon mesh filter and washed with saline until the supernatant ran clear and autoclaved for sterility. A sufficient volume was made for the planned experiments and was stored (−20°C) until use. At the time of experimentation, the gastric particles were resuspended in saline (40 mg/ml) with a pH of 1 as previously described (Nemzek et al., 2015; Raghavendran et al., 2009). Six aliquots (8 ml each) were bronchoscopically instilled to lobar bronchi 15 min following the sham renal inoculation. (4) Sham renal inoculation: A 5‐ml aliquot of phosphate buffered saline (PBS) was administered into the kidney parenchyma as described above. Intravenous crystalloids were administered starting at T_2_ (7.5–10 ml/kg/h) and continued for the duration of experimentation.

#### 2.2.3 Group 3—*combined indirect and direct lung injury*

Both direct and indirect insults were used in this group in the order of (1–2) volutrauma and hyperoxia, (3) *E. coli* renal inoculation, and (4) bronchoscopic instillation of acidified gastric particles.

### 2.3 Monitoring and data collection

Animals were monitored for at least 12 h after renal inoculation. Sequential hemodynamic measurements including MAP, PAP, HR, and temperature were monitored and recorded continuously (MP160, Biopac Inc.). Ventilation parameters including peak airway pressure (AP_peak_), respiratory rate, FiO_2_, and PetCO_2_ were recorded every hour along with arterial and mixed venous blood samples. Additional blood and and chest radiographs were obtained every 4–6 h. At the conclusion of the experiment, animals were euthanized while under general anesthesia using intravenous potassium chloride (1–2 meq/ml). Organ tissue samples were acquired for histological assessment by a pathologist with expertise in thoracic pathology. Multiple blocks from each lung were examined, and the pathologist was blinded to experimental group. Lungs were graded as “Normal,” “Possible DAD,” or “Definite DAD” based on standard criteria used to evaluate human lung tissue for DAD (Konopka et al., 2020).

The chest radiographs were scored by two blinded Pulmonary & Critical Care‐trained physicians (RPD and MWS), who rated each chest radiograph on a scale of 1–10 to quantify the extent of lung injury (1 = no abnormalities, 10 = severe, diffuse lung injury). Interobserver correlation was very high (Pearson *r* = 0.94). Chest radiograph score had high internal validity when compared to time‐matched lung injury severity (PaO_2_/FiO_2_ ratio; Figure S1).

### 2.4 Preliminary studies using sham interventions

As a pilot experiment to ensure that the above‐described instrumentation, sedation, and mechanical ventilation did not confound our primary comparisons, we subjected two pigs to “sham intervention” in which they underwent all of the described instrumentation but received an intrarenal inoculation of saline rather than *E*. *coli*. These two animals both survived for 24 h without evidence of shock or respiratory failure. PaO_2_/FiO_2_ ratio at hour 12 was 346 and 349. The histopathology of lungs (harvested at 24 h) was characterized as “normal” with no evidence of DAD.

### 2.5 Prespecified criteria for successful model development

Prior to initial experimentation, the study team agreed to prespecified criteria by which the model would be considered a successful model of human ARDS: (1) recapitulation of the physiologic and histopathologic features of human ARDS: impaired oxygenation (PaO_2_/FiO_2_ <300) and pathologic evidence of diffuse alveolar damage; (2) a time‐efficient model in which ARDS is achieved within 24 h of initial exposure. For the purposes of time‐matched comparisons, primary outcomes were PaO_2_/FiO_2_ (physiology), severity of diffuse bilateral opacities on chest radiograph (radiology), and presence of “definite diffuse alveolar damage” (histopathology).

### 2.6 Statistical analyses

Descriptive data are presented as means and standard deviation (SD). Analysis of variance with repeated measures (RM‐ANOVA) or mixed‐effects analysis (in case of missing data) were used for longitudinal analysis including a post hoc Tukey correction for multiple comparisons. Contingency testing was performed using Fisher's exact test. Interobserver correlation was determined using Pearson's correlation coefficient. Primary analysis was conducted at the 12‐hour mark. For animals that reached the prespecified stopping criteria and were euthanized prior to 12 h, their last recorded value was used. All data and figures were analyzed and created using Matlab R2017a (The MathWorks, Inc.), SAS 9.4 (version 9.4), and PRISM 8 (GraphPad Software).

## 3 RESULTS

Baseline characteristics of animals in all experimental groups are presented in Table 1. Of the 14 animals, 11 survived to 12 h for all measurements while three met prespecified criteria prior to 12 h. Two of these were in the *indirect lung injury* group (9 and 11 h of measurement) and one was in the *combined lung injury* group (11 h of measurements).

### 3.1 Oxygenation

**FIGURE 1:**
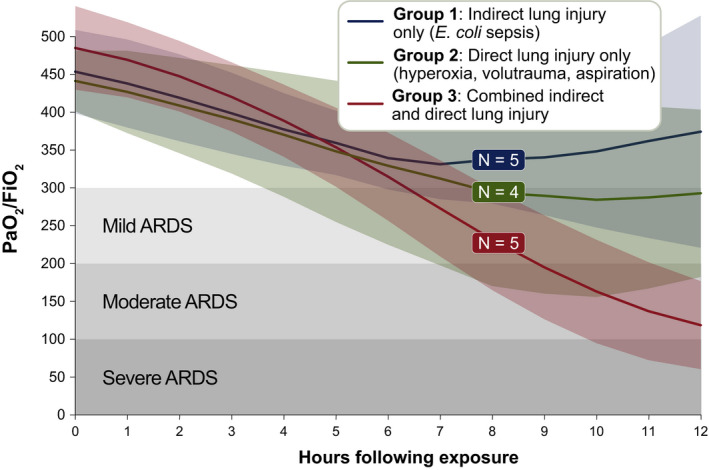
Comparison of oxygenation across experimental groups. Healthy Yorkshire‐mix swine, 14–16 weeks of age, were exposed to (1) *indirect lung injury* (*Escherichia coli* sepsis), (2) *direct lung injury* (hyperoxia, volutrauma, and aspiration of gastric particles), and (3) *combined direct and indirect lung injury* (all above exposures). Group 1 versus Group 3: *p* = 0.02. Group 2 versus Group 3: *p* = 0.03. Lines and variance represent means and standard deviation, both with Lowess smoothing. Significance determined using ANOVA with the Holm–Šidák correction for multiple comparisons

We first compared oxygenation over time across the experimental groups as assessed by PaO_2_/FiO_2_ ratio (Ranieri et al., 2012). As shown in Figure 1, experimental groups had similar baseline oxygenation. However, over time, Groups 1 (*indirect lung injury only*) and 2 (*direct lung injury only*) exhibited mild impairment in oxygenation, with mean PaO_2_/FiO_2_ plateauing at or above the definitional threshold of 300. In contrast, Group 3 (*combined indirect and direct lung injury*) exhibited a progressive decline in PaO_2_/FiO_2_ ratio from 494.1 (67.46) at baseline to 124.8 (63.80) at hour 12 (*p* = 0.0012). While within‐group variation was observed, all animals in Group 3 (*combined indirect and direct lung injury*) reached the definitional PaO_2_/FiO_2_ ratio threshold of ≤300 by hour 12. PaO_2_/FiO_2_ ratio was lower in Group 3 than either Group 1 or Group 2 (*p* = 0.02 and 0.03, respectively). We thus concluded that the *combined indirect and direct lung injury* exposures provoke a level of impaired oxygenation that is consistent with human ARDS (Ranieri et al., 2012).

### 3.2 Chest imaging

**FIGURE 2:**
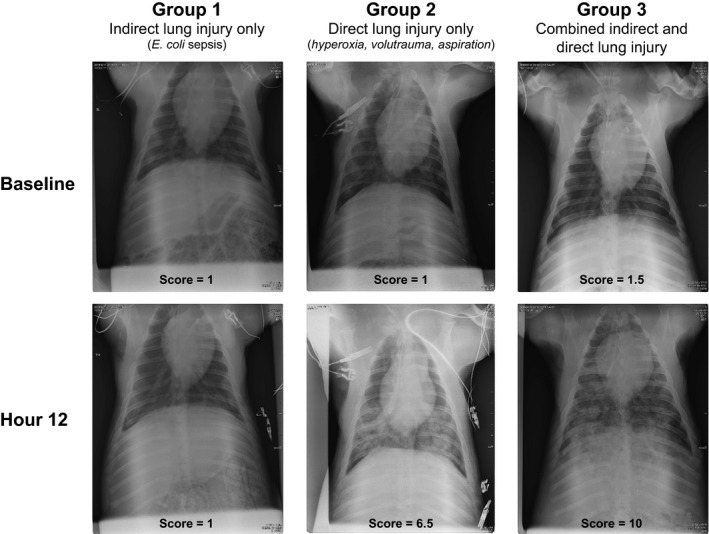
Representative chest radiographs across experimental groups. Ventral‐dorsal chest radiographs were obtained at baseline and every 4 h following exposure for the duration of the experiment. Images were scored by two Pulmonary & Critical Care Medicine physicians (blinded to experimental group and timepoint) using a scale from 1 (normal) to 10 (severe, diffuse bilateral opacities). The mean chest radiograph score across two reviewers is reported. Interobserver correlation was high (Pearson *r* = 0.94)

We next compared serial chest radiographs from the animals in each experimental group (Figure 2). We specifically assessed for the presence of bilateral opacities, another definitional feature of ARDS (Ranieri et al., 2012). Chest radiographs were scored by two Pulmonary & Critical Care‐trained physicians, blinded to experimental group and time point, using a scale of 1–10 (1 = no abnormalities, 10 = severe diffuse bilateral opacities). Chest radiographs were obtained on a single animal in Group 1 (*indirect lung injury only*); these images were scored as normal (score = 1) both at baseline at hour 12. In contrast, both Groups 2 (*direct lung injury only*) and 3 (*combined direct and indirect lung injury*) animals exhibited significant increases in chest radiograph abnormalities. In both groups, all baseline radiographs were scored as normal with a range of 1–3. In Group 2 (*direct lung injury only*), the chest radiograph score increased to a mean of 6.3 (SD 1.4) (range 4.5–8, 95% CI: 4.1, 8.6). Group 3 (*combined indirect and direct lung injury*) increased to a mean of 7.4 (SD 2.4) (range 4.5–10, 95% CI: 4.4, 10). As a test of internal validity, we compared the severity of impaired oxygen (PaO_2_/FiO_2_ ratio) and severity of injury on chest radiographs (chest radiograph severity score). Mixed effects regression confirmed that an increased chest radiograph severity score was significantly associated with decreased PaO_2_/FiO_2_ ratio when adjusted for experimental group and time point (*p* < 0.001). These data demonstrate that the *combined indirect and direct lung injury* exposures result in the development of diffuse bilateral pulmonary infiltrates that are consistent with the human ARDS definition (Ranieri et al., 2012).

### 3.3 Histopathology

**FIGURE 3:**
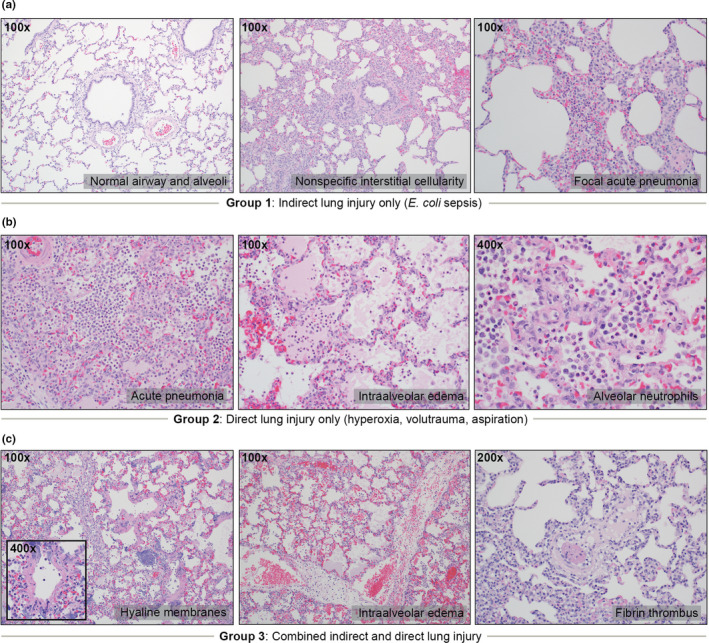
Representative histopathology across experimental groups. Post‐mortem lung tissue was examined by an expert thoracic pathologist using a semi‐quantitative instrument for identifying key features of diffuse alveolar damage (DAD). (a) Of the four animals examined in Group 1 (*indirect lung injury only*), three were graded as *normal*. Abnormal findings included mildly increased interstitial cellularity and focal acute pneumonia in a single animal. No animals in Group 1 exhibited features of DAD. (b) Of the four animals examined in Group 2 (*direct lung injury only*), all four exhibited features of acute bronchopneumonia with intra‐alveolar edema. No animals in Group 2 exhibited features of DAD. (c) Of the five animals examined in Group 3 (*combined indirect and direct lung injury*), 4/5 were classified as *definite DAD*. Prominent findings in Group 3 included hyaline membranes (4/5), intra‐alveolar edema (3/5), fibrin thrombi (5/5) and acute bronchopneumonia (5/5)

The histopathology of postmortem lung tissue from the three experimental groups was assessed by an expert thoracic pathologist (Figure 3). None of the specimens from Group 1 (*indirect lung injury only*, *n* = 4) or Group 2 (*direct lung injury*, *n* = 4) exhibited the core features of DAD, including hyaline membrane formation (the histopathological hallmark of DAD). In contrast, in Group 3 (*combined indirect and direct lung injury*, *n* = 5), lung tissue from four of five animals met criteria for *definite DAD* based on the presence of hyaline membranes and other key features (e.g., intra‐alveolar edema and fibrin thrombi) (*p* = 0.04 vs. Group 1 and Group 2). Within Group 1 (*indirect lung injury only*), three of four examined lungs were histologically graded as *normal*, with a single animal exhibiting increased interstitial cellularity and focal acute pneumonia. Within Group 2, four of four examined lungs were characterized by acute bronchopneumonia with intra‐alveolar edema. As such, the *combined indirect and direct lung injury* exposures resulted in DAD, whereas the individual *indirect lung injury* and *direct lung injury* exposures did not.

### 3.4 Physiologic, inflammatory, and extrapulmonary organ function measurements

Additional data regarding physiologic, immunologic, and extrapulmonary organ function measurements are included in the Online Supplement available at https://figshare.com/s/c6730605e540f948e317 (https://doi.org/10.6084/m9.figshare.13703365). At hour 12, peak airway pressures were increased in Group 2 (*direct lung injury only*) and Group 3 (*combined indirect and direct lung injury*) relative to Group 1 (*indirect lung injury only*) (Figure S2). Temperature was greater in Group 1 (*indirect lung injury only*) than in Group 2 or 3. Arterial carbon dioxide (PaCO_2_) was greater in Group 3 (*combined indirect and direct lung injury*) than in Group 1 (*indirect lung injury only*). Experimental groups did not differ at hour 12 in their white blood cell count or relative neutrophilia (Figure S3). Groups 1 (*indirect lung injury only*) and 3 (*combined indirect and direct lung injury*) both exhibited biochemical evidence of acute kidney injury (Figure S3).

## 4 DISCUSSION

We here report a novel swine model of ARDS that faithfully recapitulates the features of human disease using common, clinically relevant injury exposures. Our model meets our prespecified criteria for successful model development for human ARDS: (1) it recapitulates the physiologic and histopathologic features of human disease (impaired oxygenation and diffuse alveolar damage) and (2) it does so in a time‐efficient manner in which ARDS is achieved within 24 h of exposure. Our novel model offers advantages over both small animal (rodent) models as well as existing swine models that rely on clinically unrepresentative single‐hit exposures (e.g., oleic acid infusion [Dickson et al., 2011; Schuster, 1994] and surfactant washout [Russ et al., 2016]). Additionally, in alignment with NHLBI clinical research priorities, our novel preclinical model (1) uses a biologically relevant infectious exposure (inoculation of viable *E. coli*) (Tiba et al., 2020), (2) allows for the study of organ dysfunction and organ support, and (3) permits cointerventions (e.g., intravenous fluids, vasopressors, and antimicrobials). Our model thus fills an important gap in the preclinical study of ARDS, a devastating and common condition for which we lack molecular diagnostics and therapeutics.

In addition to meeting our own prespecified criteria, our model meets other established criteria for ARDS. Our model consistently provokes DAD (including hyaline membrane formation), the histopathological hallmark of human ARDS. This pathological finding is highly specific and confirms that the model's hypoxemia and radiographic opacities are not attributable to competing processes (e.g., shock, cardiogenic edema, or acute pneumonia). Our model also satisfies the clinically derived Berlin Criteria (Ranieri et al., 2012), which are typically considered inapplicable to animal models given the impracticality of assessing arterial oxygenation and chest radiographs in rodents (Matute‐Bello et al., 2011). Finally, our model fulfills criteria established by a 2011 American Thoracic Society workshop on experimental lung injury in animals (Matute‐Bello et al., 2011), in that it induces (1) severe lung injury within 24 h of exposure, (2) histologic evidence of tissue injury (e.g., hyaline membranes), (3) alteration of the alveolar capillary barrier (e.g., proteinaceous edema within the alveolar space), (4) alveolar inflammation (e.g., accumulation of alveolar neutrophils), and (5) physiologic dysfunction (e.g., hypoxemia). In aggregate, our model thus robustly satisfies pathological, clinical, and experimental criteria for ARDS.

Importantly, these criteria for modeling ARDS were only met by our *combined* exposure group (both *indirect lung injury* and *direct lung injury* exposures) and were not met by its individual constituent exposures (*indirect lung injury only* or *direct lung injury only*). These findings are congruent with recurring observations, both clinical (Sjoding et al., 2019) and experimental (Wiener‐Kronish et al., 1991), that both epithelial *and* endothelial injury are necessary to yield the full pathophysiologic and clinical manifestations of ARDS. In the contemporary era, most patients with ARDS have risk factors that represent both systemic (endothelial) pathology (e.g., sepsis or shock) as well as direct lung injury (e.g., pneumonia or aspiration) (Sjoding et al., 2019). Even SARS‐CoV‐2, a pandemic respiratory virus that causes ARDS in its most severe form, provokes both epithelial and endothelial lung injury as assessed via postmortem examination (Hariri et al., 2021; Polak et al., 2020). We believe this trend has important experimental implications, as “single‐hit exposures” (such as intratracheal endotoxin in mice) are unlikely to fully recapitulate the complex pathophysiology of human ARDS. Strong consideration should be given to leveraging combined exposures to improve the biological and clinical relevance of experimental lung injury.

When compared to common rodent models of lung injury, swine modeling of ARDS unquestionably provides greater fidelity to human disease, whether characterized histopathologically, physiologically, or radiographically. Yet on a per‐experiment basis, swine modeling is far more expensive and labor intensive than rodent experimentation. We argue that these practical considerations should not dissuade the field from embracing novel large animal models of ARDS. First, the practical advantages of rodent modeling are all related to cost and convenience (e.g., size, expense, breeding rate, and availability of inbred strains and antibodies) rather than biologic fidelity. As acknowledged by the 2011 American Thoracic Society workshop report on experimental acute lung injury, murine lungs typically do not develop the cardinal histopathological features of human ARDS and thus “cannot be expected to be identical or *perhaps even similar*” to human disease (emphasis added) (Matute‐Bello et al., 2011). These practical advantages of murine modeling are of questionable significance if the models do not recapitulate human pathology. While important anatomic differences do exist between the lungs of pigs and humans (e.g., the relative prominence of pulmonary intravascular macrophages [Dehring & Wismar, 1989; Schneberger et al., 2012]), these differences are modest compared to those between rodent and human lungs (e.g., the absence of hyaline membranes in most murine models of lung injury).

Second, small animal modeling presents its own practical limitations that severely constrain investigators' ability to study ARDS pathogenesis. These limitations include the inability to (1) readily assess hypoxemia (the core physiologic feature of ARDS), (2) perform chest imaging (a definitional feature of ARDS in humans), (3) serially sample blood or alveolar fluid (and use pre‐exposure animals as their own controls), and (4) administer highly relevant co‐exposures (e.g., mechanical ventilation, vasopressor support). Third, any thorough calculation of the cost of murine modeling must include the *opportunity cost* of decades of negative clinical trials of molecular therapies (Duggal et al., 2015; Rubenfeld, 2015), nearly all of which were motivated by promising experimental findings in rodents (Matute‐Bello et al., 2011; Semler et al., 2020). While the per‐experiment cost of large animal modeling exceeds that of murine modeling, this difference pales in comparison to the collective cost of performing human trials without reliable and informative preclinical modeling. For these reasons, the NHLBI Working Group Report on Identifying Clinical Research Priorities in Adult Pulmonary and Critical Care has specifically called for improved large animals models of ARDS to inform human trial design (Semler et al., 2020).

Fourth, while the lack of genetic models and species‐specific reagents may be a current limitation to large‐animal modeling, we believe this practical limitation is overstated and temporary. Given the agricultural importance of swine, it is unsurprising that thousands of swine‐specific antibody and ELISA kits are already commercially available (Ziegler et al., 2016). Additionally, the availability of an annotated swine genome and RNA‐Seq technology makes molecular characterization of the swine host response highly feasible (Humphray et al., 2007; Ropka‐Molik et al., 2014). Further, immunohistochemical markers of swine lungs are similar to those of humans and unlike those of mice (Meyerholz et al., 2016). Fifthly, while the cost and time required to study large animals represents a potential barrier to performing adequately powered comparisons, the ability to serially sample the blood and lungs of the same animal (thus using each animal as its own control comparison) is a tremendous experimental advantage over small animal models (as well as many human studies).

Finally, large animal modeling has proven highly informative in non‐ARDS lung diseases. As an example, our understanding of the pathophysiology of cystic fibrosis has been transformed and advanced by the development of genetically modified swine models, which (unlike murine models) faithfully recapitulate human lung pathology (Meyerholz, 2016; Stoltz et al., 2010). This long‐term investment in modeling human biology—despite the short‐term expense—has dramatically advanced our understanding of the pathophysiology of cystic fibrosis, the treatment of which has been revolutionized by the successful development of targeted molecular therapies (Meyerholz, 2016). Taken together, we believe there is strong rationale for the field investing in large animal preclinical models of ARDS.

We acknowledge that our study has several limitations that should inform future investigations. First, this pilot study used only male animals to minimize one source of biologic heterogeneity. Future studies will include both male and female animals to investigate the role of sex in the susceptibility to ARDS (Heffernan et al., 2011). Second, we did not include a full negative control arm (i.e., animals that received either sham indirect and/or direct lung injury exposures). Despite this, our *indirect lung injury only* experimental group exhibited near‐normal lung histopathology, providing reassurance that supportive care and instrumentation alone are not responsible for the ARDS pathophysiology observed in our combined exposure group. Additionally, the availability of serial sampling afforded by large animal modeling permitted us to perform within‐group comparisons to baseline (pre‐exposure) measurements for physiologic and radiographic features. Finally, as reported above in Section 2, in a pilot study of two pigs who received sham inoculation and supportive care, we found no evidence of DAD or severely impaired gas exchange following 24 h of mechanical ventilation. While we successfully met our goal of establishing a model of ARDS and determining the relative contributions of the model's constituent exposures (indirect and direct lung injury), we studied a modest number of animals in each experimental group (4–5), which limits statistical power for comparing differences with small effect sizes across cohorts. Finally, we did not design the model to test differences in survival, long‐term management, or sequelæ. In future studies, our model will serve as a foundation to test interventions such as supportive care (e.g., ventilation strategies) or pharmacotherapy.

In conclusion, we report a novel high‐fidelity swine model of ARDS provoked by common, clinically relevant injury exposures. As a controlled large animal model, it permits longitudinal measurement of physiologic, radiographic, and biochemical features of disease, as well as definitive histopathologic evaluation of lung tissue. This model fills an important preclinical gap in the study of ARDS and will facilitate translational inquiry into the pathogenesis and management of this lethal and common lung condition.

## AUTHOR'S CONTRIBUTIONS

**Conceptualization**: MHT, BMM, RPD, MWS, JAN, RCD, JSV, KAS, KRW. **Data Curation**: MHT, BMM, RPD, JAN, DCL, TLF, RCD, JSV, KAS. **Formal Analysis**: MHT, BMM, DCL, KEK, MWS, TLF, KAS. **Experiments Design and Performance**: MHT, BMM, RPD, MWS, JAN, CIC, DCL, TLF, RCD, KEK, JSV, KAS. **Experiments Performance**: MHT, BMM, RPD, JAN, KEK, CIC. **Supervision**: MHT, BMM, RPD, JAN, JSV, KAS. **Writing—Original Draft Preparation**: MHT, BMM. **Writing—Review & Editing**: MHT, BMM, RPD, MWS, JAN, CIC, DCL, TLF, RCD, KEK, JSV, KAS, KRW.

## AVAILABILITY AS PREPRINT

This manuscript has been posted online as a preprint, available at: https://www.biorxiv.org/content/10.1101/2021.01.24.427964v1

## Supporting information



Fig S1

Fig S2

Fig S3

## References

[phy214871-bib-0001] Ashbaugh, D. G. , Bigelow, D. B. , Petty, T. L. , & Levine, B. E. (1967). Acute respiratory distress in adults. Lancet, 2, 319–323. 10.1016/S0140-6736(67)90168-7 4143721

[phy214871-bib-0002] Bailey, M. , Christoforidou, Z. , & Lewis, M. C. (2013). The evolutionary basis for differences between the immune systems of man, mouse, pig and ruminants. Veterinary Immunology and Immunopathology, 152, 13–19. 10.1016/j.vetimm.2012.09.022 23078904

[phy214871-bib-0003] Bernard, G. R. , Artigas, A. , Brigham, K. L. , Carlet, J. , Falke, K. , Hudson, L. , Lamy, M. , LeGall, J. R. , Morris, A. , & Spragg, R. (1994). Report of the American‐European consensus conference on ARDS: Definitions, mechanisms, relevant outcomes and clinical trial coordination. The Consensus Committee. Intensive Care Medicine, 20, 225–232. 8014293 10.1007/BF01704707

[phy214871-bib-0004] Dawson, H. D. , Loveland, J. E. , Pascal, G. , Gilbert, J. G. , Uenishi, H. , Mann, K. M. , Sang, Y. , Zhang, J. , Carvalho‐Silva, D. , Hunt, T. , Hardy, M. , Hu, Z. , Zhao, S. H. , Anselmo, A. , Shinkai, H. , Chen, C. , Badaoui, B. , Berman, D. , Amid, C. , … Tuggle, C. K. (2013). Structural and functional annotation of the porcine immunome. BMC Genomics, 14, 332. 10.1186/1471-2164-14-332 23676093 PMC3658956

[phy214871-bib-0005] Dawson, H. D. , Smith, A. D. , Chen, C. , & Urban, J. F. Jr (2017). An in‐depth comparison of the porcine, murine and human inflammasomes; lessons from the porcine genome and transcriptome. Veterinary Microbiology, 202, 2–15. 10.1016/j.vetmic.2016.05.013 27321134

[phy214871-bib-0006] Dehring, D. J. , & Wismar, B. L. (1989). Intravascular macrophages in pulmonary capillaries of humans. American Review of Respiratory Disease, 139, 1027–1029. 10.1164/ajrccm/139.4.1027 2930062

[phy214871-bib-0007] Dickson, R. P. , Hotchkin, D. L. , Lamm, W. J. , Hinkson, C. , Pierson, D. J. , Glenny, R. W. , & Rubinson, L. (2011). A porcine model for initial surge mechanical ventilator assessment and evaluation of two limited‐function ventilators. Critical Care Medicine, 39, 527–532. 10.1097/CCM.0b013e318206b99b 21187747 PMC3683595

[phy214871-bib-0008] Duggal, A. , Ganapathy, A. , Ratnapalan, M. , & Adhikari, N. K. (2015). Pharmacological treatments for acute respiratory distress syndrome: Systematic review. Minerva Anestesiologica, 81, 567–588. 24937499

[phy214871-bib-0009] Erickson, S. E. , Martin, G. S. , Davis, J. L. , Matthay, M. A. , Eisner, M. D. , & Network, N. N. A. (2009). Recent trends in acute lung injury mortality: 1996–2005. Critical Care Medicine, 37, 1574–1579. 19325464 10.1097/CCM.0b013e31819fefdf PMC2696257

[phy214871-bib-0010] Hariri, L. P. , North, C. M. , Shih, A. R. , Israel, R. A. , Maley, J. H. , Villalba, J. A. , Vinarsky, V. , Rubin, J. , Okin, D. A. , Sclafani, A. , Alladina, J. W. , Griffith, J. W. , Gillette, M. A. , Raz, Y. , Richards, C. J. , Wong, A. K. , Ly, A. , Hung, Y. P. , Chivukula, R. R. , … Mino‐Kenudson, M. (2021). Lung histopathology in coronavirus disease 2019 as compared with severe acute respiratory syndrome and H1N1 influenza: A systematic review. Chest, 159, 73–84. 33038391 10.1016/j.chest.2020.09.259 PMC7538870

[phy214871-bib-0011] Heffernan, D. S. , Dossett, L. A. , Lightfoot, M. A. , Fremont, R. D. , Ware, L. B. , Sawyer, R. G. , & May, A. K. (2011). Gender and acute respiratory distress syndrome in critically injured adults: A prospective study. Journal of Trauma, 71, 878–883; discussion 883–875. 10.1097/TA.0b013e31822c0d31 PMC3201740 21986736

[phy214871-bib-0012] Humphray, S. J. , Scott, C. E. , Clark, R. , Marron, B. , Bender, C. , Camm, N. , Davis, J. , Jenks, A. , Noon, A. , Patel, M. , Sehra, H. , Yang, F. , Rogatcheva, M. B. , Milan, D. , Chardon, P. , Rohrer, G. , Nonneman, D. , de Jong, P. , Meyers, S. N. , … Rogers, J. (2007). A high utility integrated map of the pig genome. Genome Biology, 8, R139. 10.1186/gb-2007-8-7-r139 17625002 PMC2323232

[phy214871-bib-0013] Judge, E. P. , Hughes, J. M. , Egan, J. J. , Maguire, M. , Molloy, E. L. , & O'Dea, S. (2014). Anatomy and bronchoscopy of the porcine lung. A model for translational respiratory medicine. American Journal of Respiratory Cell and Molecular Biology, 51, 334–343. 10.1165/rcmb.2013-0453TR 24828366

[phy214871-bib-0014] Konopka, K. E. , Nguyen, T. , Jentzen, J. M. , Rayes, O. , Schmidt, C. J. , Wilson, A. M. , Farver, C. F. , & Myers, J. L. (2020). Diffuse alveolar damage (DAD) resulting from coronavirus disease 2019 infection is morphologically indistinguishable from other causes of DAD. Histopathology, 77, 570–578. 10.1111/his.14180 32542743 PMC7323403

[phy214871-bib-0015] Matute‐Bello, G. , Downey, G. , Moore, B. B. , Groshong, S. D. , Matthay, M. A. , Slutsky, A. S. , & Kuebler, W. M. (2011). An official American Thoracic Society workshop report: Features and measurements of experimental acute lung injury in animals. American Journal of Respiratory Cell and Molecular Biology, 44, 725–738. 21531958 10.1165/rcmb.2009-0210ST PMC7328339

[phy214871-bib-0016] Merrifield, C. A. , Lewis, M. , Claus, S. P. , Beckonert, O. P. , Dumas, M. E. , Duncker, S. , Kochhar, S. , Rezzi, S. , Lindon, J. C. , Bailey, M. , Holmes, E. , & Nicholson, J. K. (2011). A metabolic system‐wide characterisation of the pig: A model for human physiology. Molecular BioSystems, 7, 2577–2588. 21761043 10.1039/c1mb05023k

[phy214871-bib-0017] Mestas, J. , & Hughes, C. C. (2004). Of mice and not men: Differences between mouse and human immunology. The Journal of Immunology, 172, 2731–2738. 14978070 10.4049/jimmunol.172.5.2731

[phy214871-bib-0018] Meurens, F. , Summerfield, A. , Nauwynck, H. , Saif, L. , & Gerdts, V. (2012). The pig: A model for human infectious diseases. Trends in Microbiology, 20, 50–57. 22153753 10.1016/j.tim.2011.11.002 PMC7173122

[phy214871-bib-0019] Meyerholz, D. K. (2016). Lessons learned from the cystic fibrosis pig. Theriogenology, 86, 427–432. 10.1016/j.theriogenology.2016.04.057 27142487 PMC4885762

[phy214871-bib-0020] Meyerholz, D. K. , Lambertz, A. M. , Reznikov, L. R. , Ofori‐Amanfo, G. K. , Karp, P. H. , McCray, P. B. Jr , Welsh, M. J. , & Stoltz, D. A. (2016). Immunohistochemical detection of markers for translational studies of lung disease in pigs and humans. Toxicologic Pathology, 44, 434–441. 10.1177/0192623315609691 26511846 PMC4805467

[phy214871-bib-0021] National Research Council (U.S.) Committee for the Update of the Guide for the Care and Use of Laboratory Animals, Institute for Laboratory Animal Research (U.S.), and National Academies Press (U.S.) . (2011). Guide for the care and use of laboratory animals. National Academies Press.

[phy214871-bib-0022] Nemzek, J. A. , Hodges, A. P. , & He, Y. (2015). Bayesian network analysis of multi‐compartmentalized immune responses in a murine model of sepsis and direct lung injury. BMC Research Notes, 8, 516. 10.1186/s13104-015-1488-y 26423575 PMC4589912

[phy214871-bib-0023] Polak, S. B. , Van Gool, I. C. , Cohen, D. , von der Thüsen, J. H. , & van Paassen, J. (2020). A systematic review of pathological findings in COVID‐19: A pathophysiological timeline and possible mechanisms of disease progression. Modern Pathology, 33, 2128–2138. 32572155 10.1038/s41379-020-0603-3 PMC7306927

[phy214871-bib-0024] Raghavendran, K. , Davidson, B. A. , Huebschmann, J. C. , Helinski, J. D. , Hutson, A. D. , Dayton, M. T. , Notter, R. H. , & Knight, P. R. (2009). Superimposed gastric aspiration increases the severity of inflammation and permeability injury in a rat model of lung contusion. Journal of Surgical Research, 155, 273–282. 10.1016/j.jss.2008.08.020 PMC2716411 19515386

[phy214871-bib-0025] Ranieri, V. M. , Rubenfeld, G. D. , Thompson, B. T. , Ferguson, N. D. , Caldwell, E. , Fan, E. , Camporota, L. , & Slutsky, A. S. (2012). Acute respiratory distress syndrome: The Berlin Definition. JAMA, 307, 2526–2533. 22797452 10.1001/jama.2012.5669

[phy214871-bib-0026] Robles, A. J. , Kornblith, L. Z. , Hendrickson, C. M. , Howard, B. M. , Conroy, A. S. , Moazed, F. , Calfee, C. S. , Cohen, M. J. , & Callcut, R. A. (2018). Health care utilization and the cost of posttraumatic acute respiratory distress syndrome care. Journal of Trauma and Acute Care Surgery, 85, 148–154. 10.1097/TA.0000000000001926 PMC6029709 29958249

[phy214871-bib-0027] Ropka‐Molik, K. , Zukowski, K. , Eckert, R. , Gurgul, A. , Piórkowska, K. , & Oczkowicz, M. (2014). Comprehensive analysis of the whole transcriptomes from two different pig breeds using RNA‐Seq method. Animal Genetics, 45, 674–684. 10.1111/age.12184 24961663

[phy214871-bib-0028] Rubenfeld, G. D. (2015). Confronting the frustrations of negative clinical trials in acute respiratory distress syndrome. Annals of the American Thoracic Society, 12(Suppl 1), S58–S63. 10.1513/AnnalsATS.201409-414MG 25830838

[phy214871-bib-0029] Russ, M. , Kronfeldt, S. , Boemke, W. , Busch, T. , Francis, R. C. , & Pickerodt, P. A. (2016). Lavage‐induced surfactant depletion in pigs as a model of the acute respiratory distress syndrome (ARDS). Journal of Visualized Experiments, 115, 53610. 10.3791/53610 PMC5091986 27684585

[phy214871-bib-0030] Schneberger, D. , Aharonson‐Raz, K. , & Singh, B. (2012). Pulmonary intravascular macrophages and lung health: What are we missing? American Journal of Physiology. Lung Cellular and Molecular Physiology, 302, L498–L503. 10.1152/ajplung.00322.2011 22227203

[phy214871-bib-0031] Schuster, D. P. (1994). ARDS: Clinical lessons from the oleic acid model of acute lung injury. American Journal of Respiratory and Critical Care Medicine, 149, 245–260. 8111590 10.1164/ajrccm.149.1.8111590

[phy214871-bib-0032] Semler, M. W. , Bernard, G. R. , Aaron, S. D. , Angus, D. C. , Biros, M. H. , Brower, R. G. , Calfee, C. S. , Colantuoni, E. A. , Ferguson, N. D. , Gong, M. N. , Hopkins, R. O. , Hough, C. L. , Iwashyna, T. J. , Levy, B. D. , Martin, T. R. , Matthay, M. A. , Mizgerd, J. P. , Moss, M. , Needham, D. M. , … Reineck, L. A. (2020). Identifying clinical research priorities in adult pulmonary and critical care: NHLBI Working Group Report. American Journal of Respiratory and Critical Care Medicine, 202(4), 511–523. 10.1164/rccm.201908-1595WS PMC7427373 32150460

[phy214871-bib-0033] Sjoding, M. W. , Hofer, T. P. , Co, I. , McSparron, J. I. , & Iwashyna, T. J. (2019). Differences between patients in whom physicians agree and disagree about the diagnosis of acute respiratory distress syndrome. Annals of the American Thoracic Society, 16, 258–264. 10.1513/AnnalsATS.201806-434OC 30321489 PMC6376946

[phy214871-bib-0034] Stoltz, D. A. , Meyerholz, D. K. , Pezzulo, A. A. , Ramachandran, S. , Rogan, M. P. , Davis, G. J. , Hanfland, R. A. , Wohlford‐Lenane, C. , Dohrn, C. L. , Bartlett, J. A. , Nelson, G. A. 4th , Chang, E. H. , Taft, P. J. , Ludwig, P. S. , Estin, M. , Hornick, E. E. , Launspach, J. L. , Samuel, M. , Rokhlina, T. , … Welsh, M. J. (2010). Cystic fibrosis pigs develop lung disease and exhibit defective bacterial eradication at birth. Science Translational Medicine, 2, 29ra31. 10.1126/scitranslmed.3000928 PMC2889616 20427821

[phy214871-bib-0035] Tiba, M. H. , McCracken, B. M. , Dickson, R. P. , Nemzek, J. A. , Colmenero, C. I. , Leander, D. C. , Flott, T. L. , Daniels, R. C. , Konopka, K. E. , VanEpps, J. S. , Stringer, K. A. , & Ward, K. R. (2020). A comprehensive assessment of multi‐system responses to a renal inoculation of uropathogenic *E. coli* in swine. PLoS One, 15, e0243577. 10.1371/journal.pone.0243577 33306742 PMC7732124

[phy214871-bib-0036] Wernersson, R. , Schierup, M. H. , Jorgensen, F. G. , Gorodkin, J. , Panitz, F. , Staerfeldt, H. H. , Christensen, O. F. , Mailund, T. , Hornshoj, H. , Klein, A. , Wang, J. , Liu, B. , Hu, S. , Dong, W. , Li, W. , Wong, G. K. , Yu, J. , Wang, J. , Bendixen, C. , … Bolund, L. (2005). Pigs in sequence space: A 0.66X coverage pig genome survey based on shotgun sequencing. BMC Genomics, 6, 70. 15885146 10.1186/1471-2164-6-70 PMC1142312

[phy214871-bib-0037] Wiener‐Kronish, J. P. , Albertine, K. H. , & Matthay, M. A. (1991). Differential responses of the endothelial and epithelial barriers of the lung in sheep to *Escherichia coli* endotoxin. Journal of Clinical Investigation, 88, 864–875. 10.1172/JCI115388 PMC295473 1885774

[phy214871-bib-0038] Ziegler, A. , Gonzalez, L. , & Blikslager, A. (2016). Large animal models: The key to translational discovery in digestive disease research. Cellular and Molecular Gastroenterology and Hepatology, 2, 716–724. 28090566 10.1016/j.jcmgh.2016.09.003 PMC5235339

