# Supplementary material for: A novel swine model of the acute respiratory distress syndrome using clinically relevant injury exposures
Source: Physiol Rep. 2021 May 15;9(9):e14871. doi: 10.14814/phy2.14871 (PMC8123544; doi:10.14814/phy2.14871)
Supplement: Supplementary file 1 — Fig S1 [file PHY2-9-e14871-s002.pdf]

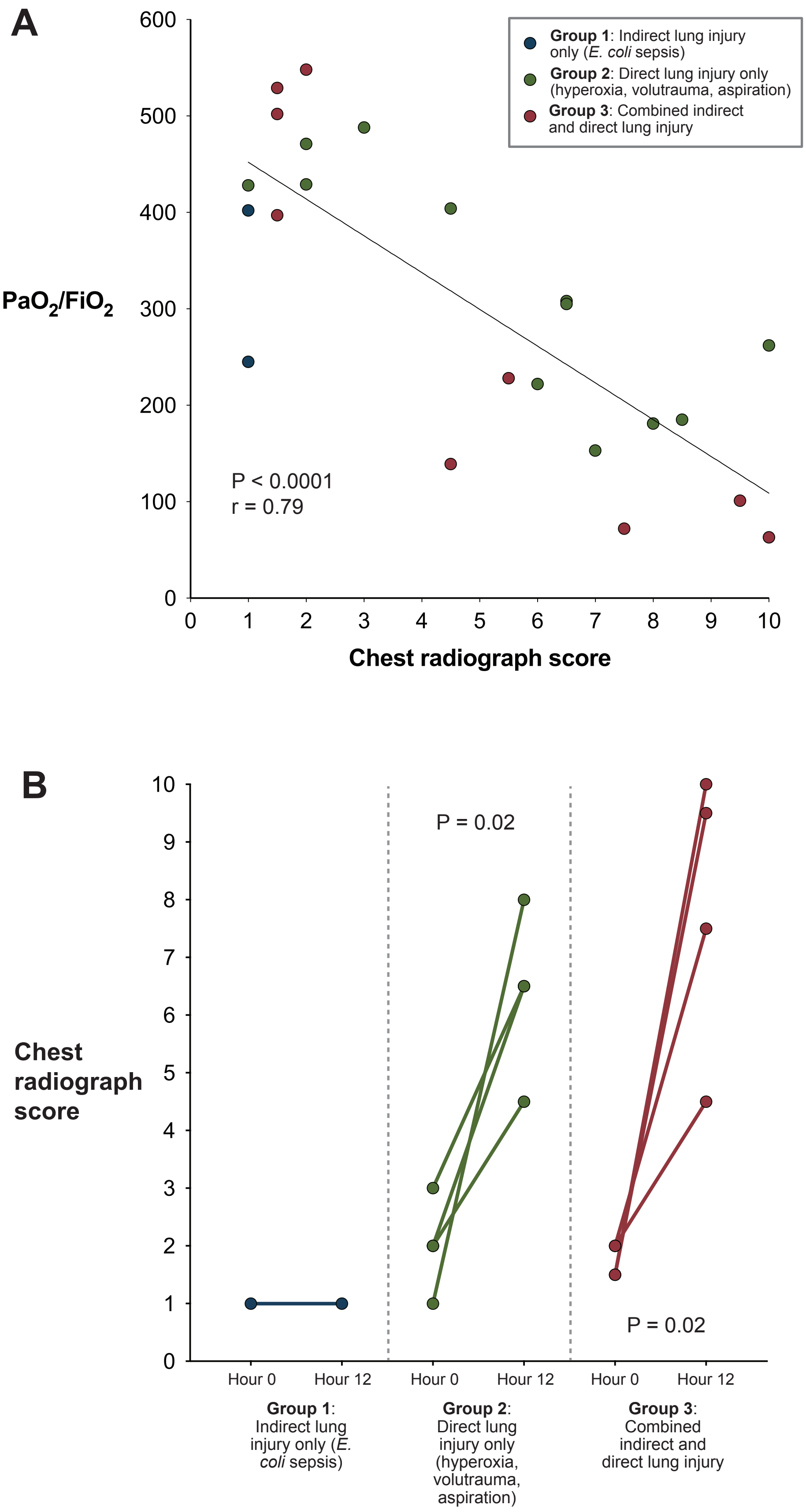

Supplemental Figure 1. Chest radiograph scores across experimental groups. Healthy Yorkshire-mix swine, 14-16 weeks of age, were exposed to 1) *indirect lung injury* (*E. coli* sepsis), 2) *direct lung injury* (hyperoxia, volutrauma, and aspiration of gastric particles), and 3) *combined direct and indirect lung injury* (all above exposures). Chest radiographs were taken at multiple timepoints and scored by two blinded Pulmonary and Critical Care Medicine physicians for severity of diffuse bilateral opacities (1-10). Interobserver correlation for chest radiograph score was high (Pearson r = 0.93). (A) Chest radiograph score correlated with physiologic severity of lung injury (PaO_2/FiO_2). (B) Chest radiograph score was increased at hour 12 relative to hour 0 in Group 2 (*direct lung injury*) and Group 3 (*combined direct and indirect lung injury*). A: Pearson r test. B: paired t-test.
