# Supplementary material for: A novel swine model of the acute respiratory distress syndrome using clinically relevant injury exposures
Source: Physiol Rep. 2021 May 15;9(9):e14871. doi: 10.14814/phy2.14871 (PMC8123544; doi:10.14814/phy2.14871)
Supplement: Supplementary file 3 — Fig S3 [file PHY2-9-e14871-s003.pdf]

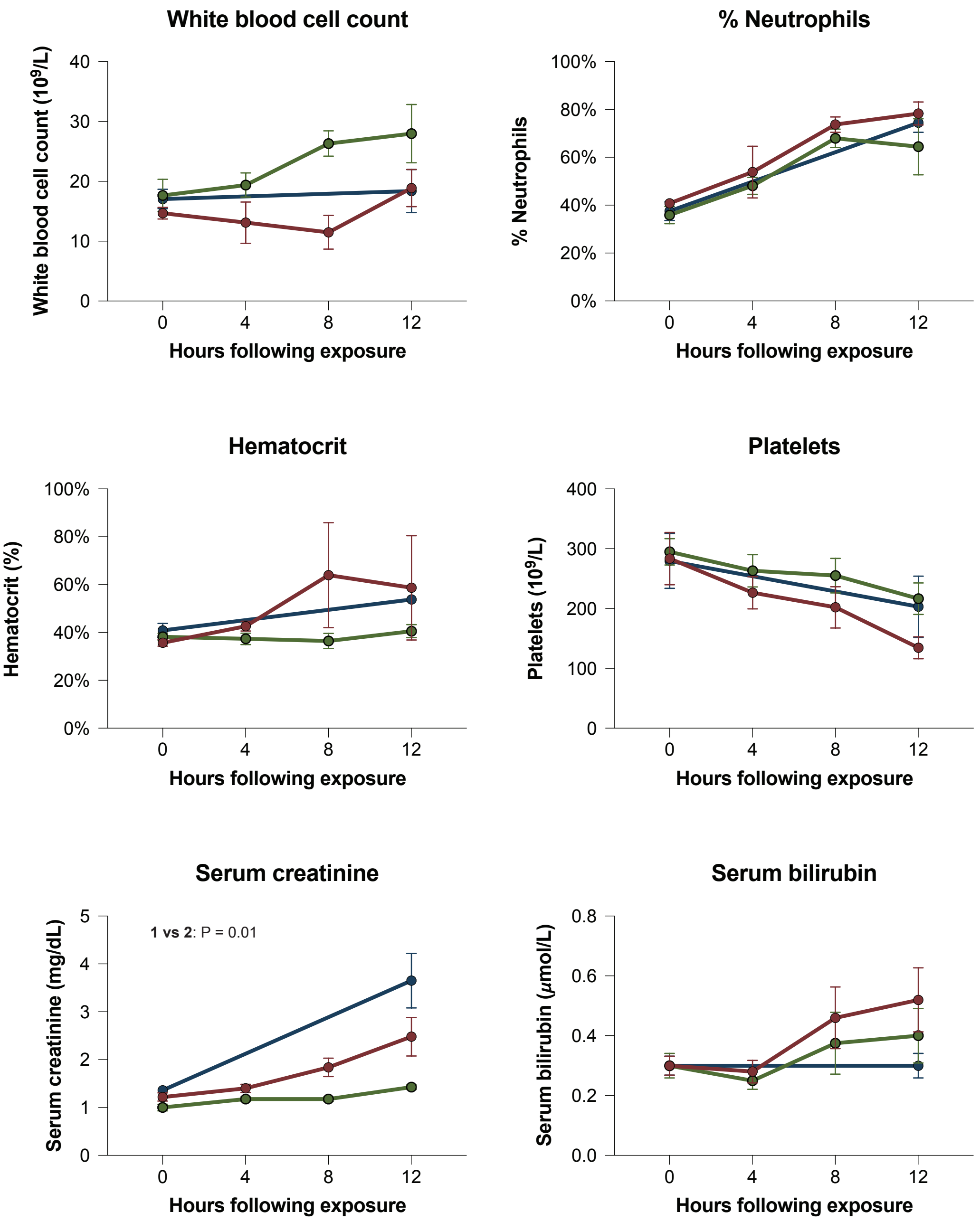

Group 1: Indirect lung injury only (*E. coli* sepsis)

Group 2: Direct lung injury only (hyperoxia, volutrauma, aspiration)

Group 3: Combined indirect and direct lung injury

Supplemental Figure 3. Comparison of laboratory values across experimental groups. Healthy Yorkshire-mix swine, 14-16 weeks of age, were exposed to 1) *indirect lung injury* (*E. coli* sepsis), 2) *direct lung injury* (hyperoxia, volutrauma, and aspiration of gastric particles), and 3) *combined direct and indirect lung injury* (all above exposures). Lines and variance represent means and standard deviation. Significance determined using ANOVA with Tukey's multiple comparisons test. When not reported, P > 0.05.
